# Winter survival of the unicellular green alga *Micrasterias denticulata*: insights from field monitoring and simulation experiments

**DOI:** 10.1007/s00709-021-01682-6

**Published:** 2021-07-24

**Authors:** Philip Steiner, Othmar Buchner, Ancuela Andosch, Andreas Holzinger, Ursula Lütz-Meindl, Gilbert Neuner

**Affiliations:** 1 Department of Biosciences, University of Salzburg, Hellbrunnerstrasse 34, 5020 Salzburg, Austria; 2 Present Address: Institute of Pharmacology, University of Linz, Huemerstrasse 3-5, 4020 Linz, Austria; 3 Department of Botany, Functional Plant Biology, University of Innsbruck, Sternwartestrasse 15, 6020 Innsbruck, Austria

**Keywords:** Cold acclimation, Golgi bodies, Endoplasmic reticulum, Freezing tolerance, Mucilage vesicles, Ultrastructure

## Abstract

Peat bog pools around Tamsweg (Lungau, Austria) are typical habitats of the unicellular green alga *Micrasterias denticulata*. By measurement of water temperature and irradiation throughout a 1-year period (2018/2019), it was intended to assess the natural environmental strain in winter. Freezing resistance of *Micrasterias* cells and their ability to frost harden and become tolerant to ice encasement were determined after natural hardening and exposure to a cold acclimation treatment that simulated the natural temperature decrease in autumn. Transmission electron microscopy (TEM) was performed in laboratory-cultivated cells, after artificial cold acclimation treatment and in cells collected from field. Throughout winter, the peat bog pools inhabited by *Micrasterias* remained unfrozen. Despite air temperature minima down to −17.3 °C, the water temperature was mostly close to +0.8 °C. The alga was unable to frost harden, and upon ice encasement, the cells showed successive frost damage. Despite an unchanged freezing stress tolerance, significant ultrastructural changes were observed in field-sampled cells and in response to the artificial cold acclimation treatment: organelles such as the endoplasmic reticulum and thylakoids of the chloroplast showed distinct membrane bloating. Still, in the field samples, the Golgi apparatus appeared in an impeccable condition, and multivesicular bodies were less frequently observed suggesting a lower overall stress strain. The observed ultrastructural changes in winter and after cold acclimation are interpreted as cytological adjustments to winter or a resting state but are not related to frost hardening as *Micrasterias* cells were unable to improve their freezing stress tolerance.

## Introduction

For half a century, the unicellular green alga *Micrasterias denticulata* has been used as a common cell biological research object (Kiermayer 1981; Lütz-Meindl 2016; Meindl 1993). Due to its close phylogenetic relationship to higher plants (Cheng et al. 2019; Leliaert et al. 2012), the species is particularly valuable in an evolutionary context. Beyond cell biological studies, *Micrasterias* has become a convenient model organism for addressing molecular and stress physiological questions (Affenzeller et al. 2009; Giddings et al. 1980; Kiermayer 1968; Lütz-Meindl 2016; Meindl 1993; and more). Only recently, by means of high-resolution 2-D and 3-D electron microscopy, it was shown that *Micrasterias* cells aggregate and fuse their mitochondria to local networks during chilling and freezing stress, assuming to be important for low-temperature stress management (Steiner et al. 2020). Despite these extensive studies, (1) only little is known under which environmental conditions *Micrasterias* survives winter and (2) if it tolerates ice encasement or can frost harden. (3) It is currently unknown if *Micrasterias* cells, when field-sampled during winter, show similar ultrastructural changes as found after cold acclimation treatment in the laboratory (Steiner et al. 2020).

For higher land plants, mechanisms of freezing survival and occasionally also the freezing strain occurring in their natural habitat are quite well-known (Buchner and Neuner 2011; Kuprian et al. 2014; Neuner et al. 2020; Sakai and Larcher 1987; Stegner et al. 2020). While trees get fully exposed to freezing air temperatures, most other species escape cold winter temperatures by soil burial (Sakai and Larcher 1987), snow coverage (Neuner et al. 1999a) or in case of hydrophytes in the water body (Larcher 1983). Environmental conditions under which green algae, representing poikilohydric plants, survive winter are less investigated, but aquatic algae likely escape freezing by staying in the water body (Hampton et al. 2017). *Micrasterias* cells react similarly but rather settle at the ground, epiphytically or on debris. Freezing may only get an issue in the interface areas, where algae might be enclosed into ice. There are some reports on frost survival of algae from laboratory and field observations (Frenette et al. 2008; Terumoto 1967; Vishnivetskaya et al. 2004), and also members of the Zygnemataceae showed freezing tolerance (Hawes 1990; Pichrtova et al. 2014). An experimental study determined the LT_50_ value for young *Zygnema* sp. cultures between −5.9 and −6.5 °C and for pre-akinetes even at −26.6 °C (Trumhova et al. 2019). However, until now, ecophysiological aspects of winter survival in *Micrasterias* have not been investigated, and environmental parameters allowing winter survival have not been measured.

Little is known about how algae cold acclimate, i.e. metabolic adjustments and development of higher resistance to freezing (Kacperslda 2000). Freezing-tolerant algae show often also a significant resistance to desiccation stress (Holzinger and Karsten 2013; Holzinger et al. 2011; Remias and Lütz 2005). This is not surprising, since contact to extracellular ice in tissues of higher plants is known to act freeze dehydrating on cells (Arora 2018). The dehydration force is a steep water potential gradient from the extracellular ice to supercooled cellular water (Mazur 1963) that becomes more pronounced the lower the freezing temperature is (Rajashekar and Burke 1996). Ice encasement of unicellular algae may act in a similar way. *Micrasterias* is a freshwater alga and inhabits peat bogs. As an aquatic organism with low osmolarity, it does not tolerate desiccation stress. Although currently unknown, this may rather indicate low tolerance to ice encasement and low freezing resistance of *Micrasterias* cells.

In previous studies, cellular ultrastructure of *Micrasterias* has been studied in cells, cultured under standard laboratory conditions (see also Andosch et al. 2012; Niedermeier et al. 2018; Steiner et al. 2020; and more). In a recent study, significant ultrastructural changes in *Micrasterias* cells were reported in response to chilling and freezing stress (Steiner et al. 2020). To the best of our knowledge, ultrastructure of field-sampled cells of *Micrasterias* has not been studied, in particular of samples taken during the winter period. However, ultrastructural changes have been reported in the closely related desmid *Cosmarium*, field-sampled in response to chilling stress at +0.6 °C (Stamenkovic et al. 2014). While for higher plants various cellular changes during winter are pretty well documented (Larcher 2003), it is not known if *Micrasterias* cells show specific ultrastructural modifications in response to natural winter conditions.

We hypothesize that (A) in peat bog pools inhabited by *Micrasterias* water remains unfrozen allowing the cells to escape freezing throughout winter. (B) We suggest that *Micrasterias* cells cannot survive ice encasement and are not able to frost harden. Furthermore, we hypothesize that (C) *Micrasterias* cells, when harvested in late autumn, should show similar ultrastructural modifications as found after cold acclimation treatment in the laboratory. In order to test these hypotheses, we performed the following experiments: (1) in a natural peat bog pool site inhabited by *Micrasterias*, microclimate was recorded throughout a whole year (2018 to 2019), and (2) testing of the algae’s ability in tolerating ice encasement and to frost harden was done by exposure to controlled low-temperature treatments. (3) Transmission electron microscopic investigations of artificially cold acclimated cells and cells sampled in the field in late autumn after natural cold acclimation were performed in comparison to that of *Micrasterias* cells cultured at +20 °C under standard laboratory conditions. This data set should provide comprehensive insights into natural winter and growth conditions, adaptation and stress management strategies and into subcellular modifications triggered by cold acclimation.

## Material and methods

All applied chemicals in this study were acquired from Sigma Aldrich (Vienna, Austria) and Roth (Karlsruhe, Germany) unless stated differently.

### Microclimate and study site

The study site was located near St. Margarethen in Lungau, Tamsweg, Salzburg, Austria (“Saumoos”), at 1050 m a.s.l (47°05’N/13°14’E). The vegetation of the peat bog “Saumoos” has been described in detail in a historic study (Bersch and Zailer 1902). The mountain pine peat bog is approximately 7000–10000 years old and originates from a flooding bog. It was used economically (peat digging) until 1966 and has been successfully re-watered and renatured since 2009. The measurements of the microclimate took place in the immediate vicinity of a pronounced and Desmidiaceae rich bend at the foot of an approximately 1 m high south-western peat cutting edge. From this location, *Micrasterias* was sampled for subsequent cultivation under laboratory conditions. All experiments were carried out with these cultures, except for the transmission electron microscopy (TEM) investigations of the field-sampled cells, which were chemically fixed on site in the forenoon on October 17th, 2018.

Microclimate data were collected from August 1st, 2018, to July 31st, 2019. Data were recorded with a solar-powered climate station consisting of a programmable data logger (CR1000, Campbell Scientific, Loughborough, UK), temperature sensors (thermocouple wire; Type-T, GG-Ti-28, Omega Engineering Inc., Stamford, CT) and a quantum sensor (QS, Delta-T, Cambridge, UK). The recording interval was set at 30 min, and the information was automatically sent twice a day by a 4G modem to a local server at the University of Salzburg in order to continuously check the integrity of the station. In addition to water temperature (8 ± 2 cm below the water surface), air temperature (standard height, 2 m) and photosynthetic photon flux density (PPFD; 5 cm above the water level) were recorded. From these data, various daily and seasonal winter minimum, maximum and mean temperatures, amplitudes and rates of temperature change were calculated. Due to an interruption of the power supply from November 4th to November 10th, 2018, we lost the data during this period.

### Cultivation of *Micrasterias*

Cultivation of *Micrasterias denticulata* Bréb., further designated as laboratory conditions (LC), was carried out in Erlenmeyer flasks with 30 ml of Desmidiaceaen medium (Schlösser 1982). Cells were cultured in a climate chamber at +20 °C and under a light/dark cycle of 14/10 h. Irradiation intensity was between 100 and 150 μmol photons m^-2^∙s^-1^. Sub-cultivation of *Micrasterias* cells was carried out every 3 to 4 weeks.

### Temperature effect on cell division rates

To obtain information on the effects of low temperature on growth, cell division rates (CDR) were assessed. A defined number of LC cultivated interphase cells of *Micrasterias* were collected and exposed to different constant temperatures: +4, +10, +15 and +20 °C. The number of cells was counted regularly during 21 days. Each experimental approach were repeated 3 times with 9 biological replicates. The data was used to calculate average CDR as a percentage increase in the cell number per day, based on the current cell number (1):

1 $$ CDR=100\%\cdotp \frac{n\left(t2\left)-n\right(t1\right)}{n(t1)\cdotp n(d)} $$

where:

n(t1): is the number of cells at time t1
n(t2): is the number of cells at time t2
n(d): is the number of days (t2-t1)

### Freezing resistance and tolerance to ice encasement

*Micrasterias* cells (LC) were transferred into Eppendorf tubes (2 ml) and were cooled in an automatic freezing unit (AFU; Buchner et al. 2020) at a cooling rate of −8 °C·h^-1^ from +20 °C to +4 °C and at −2 °C·h^-1^ from +4 °C to different target freezing temperatures: −2, −4, −6 and −8 °C. Different exposure durations (1 to 123 h) were tested. The cooling rates were chosen on the basis of tests, already carried out on freezing resistance of algae (Nagao et al. 2008; Šabacká and Elster 2006) and in accordance with cooling rates, as generally used for testing frost resistance of plants (Buchner and Neuner 2010; Larcher et al. 1990; Neuner et al. 2020; Sierra-Almeida et al. 2009). Temperature was monitored by small thermocouple sensors (see above), which were attached to the Eppendorf tubes. During exposure to freezing temperatures, samples were either kept ice-free in the supercooled state or artificially ice inoculated by transferring small amounts of ice crystals to the samples. This ensured a uniform starting temperature for freezing of the Desmidiaceaen medium and the resulting ice encasement of the algae. By this, tolerance to ice encasement was assessed. At the end of the freezing treatment, samples were carefully thawed at room temperature. Every test was repeated 3 times with 5 biological replicates each.

Viability of *Micrasterias* cells was tested after exposure to freezing temperature stress by the plasmolysis test (for method, see Andosch et al. 2012). Viability was calculated by relating the number of living cells to the total number of cells and expressing the results as a percentage. Also, for each exposure temperature, the time period after which ice encasement led to the death of 50% of the cells (Ld_50_; lethal duration) was determined by fitting a logistic function into the data (Fig. P V.2.7 Software, Biosoft, Durham, USA).

### Experimental cold acclimation

**Fig. 1:**
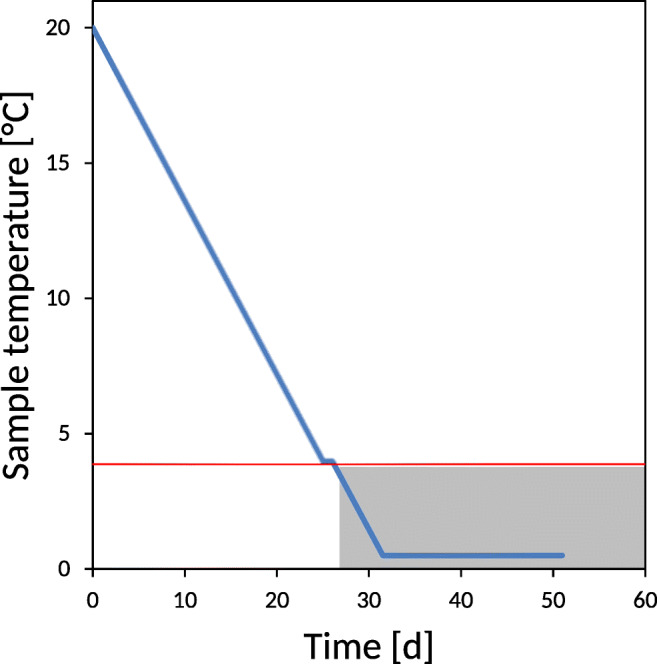
Temperature and irradiation conditions during the cold acclimation treatment. Cooling was performed at a rate of −0.6 °C·d^-1^ and under low light (25 μMol photons m^-2^·s^-1^, 8 h per day). When +4 °C (red line) was reached, the samples were darkened (grey box) and further slowly cooled down (−0.6 °C·d^-1^) to +0.5 °C and exposed for 26 days

In order to simulate natural drop of temperatures in autumn, in an artificial laboratory-based test, LC cultured *Micrasterias* cells were exposed to a long-term cold acclimation treatment lasting for 51 days (Fig. 1). The cells were initially slowly cooled down to +4 °C at a rate of −0.6 °C·d^-1^ under low irradiation intensity (25 μMol photons m^-2^·s^-1^, 8 h per day). Then, the cells were darkened as occurs by snow coverage in winter and cooled further down to +0.5 °C again at −0.6 °C·d^-1^. Darkness, the slow cooling rate and the target temperature were chosen based on micrometeorological observations made during the transition from autumn to winter at the “Saumoos” site. Freezing resistance and ice encasement tolerance were tested before and after this cold acclimation treatment to assess the cold acclimation capacity and potential changes in ice encasement tolerance.

### Transmission electron microscopy

*Micrasterias* control cells kept at +20 °C (LC) and cells kept at +0.5 °C after 51 days experimental cold acclimation (see above) were packed in cotton fibres (Meindl et al. 1992) and were transferred into gold specimen holders for subsequent high-pressure freezing (HPF). The HPF procedure was carried out in a Leica EMPACT HPF device (Leica Microsystems, Vienna, Austria) at 2040 bar and cooling rates of –12000 °C∙s^-1^. After cryosubstitution (2% OsO_4_, 0.05% uranyl acetate in anhydrous acetone) with a Leica EM AFS (Leica Microsystems, Vienna, Austria), samples were embedded in epoxy resin and prepared for TEM as previously described (Aichinger and Lütz-Meindl 2005; Steiner et al. 2018).

In order to preserve field samples directly after collection in autumn (October 17th, 2018), when temperatures were already decreasing, chemical fixation of *Micrasterias* was carried out directly in the field with 1 % glutaraldehyde (in 50 mM cacodylate buffer) for 10 min and after washing in buffer followed by 2% osmium tetroxide for 24 h. Cells were then dehydrated in ethanol steps and were embedded in epoxy resin (for detailed method, see Meindl 1983). The standard chemical fixation protocol carried out for field samples of *Micrasterias* is routinely carried out also in other desmids (Stamenkovic et al. 2014) collected in remote locations (e.g. Rippin et al. 2019) when no access to an HPF device is given. A transfer of field samples to the laboratory for HPF would certainly have more influence on the ultrastructure than chemical fixation on site. As possible artefacts of a chemical fixation are well described, a comparison with HPF was possible, not least because of the early investigations on *Micrasterias* that used chemical fixation (Meindl 1983). When HPF fixation was introduced for laboratory-grown cells, a superior fixation quality was achieved (Aichinger and Lütz-Meindl 2005; Niedermeier et al. 2018; and more), which does not mean that the here instigated structures cannot be found or interpreted in chemically fixed cells.

TEM investigations were performed with a LEO 912 AB Omega TEM (Zeiss, Oberkochen, Germany) with an accelerating voltage of 80 kV. TEM micrographs were always filtered at zero energy loss. Recording of images was obtained by a TRS 2k Slow-Scan CCD camera (Tröndle Restlicht Verstärker Systeme, Moorenweis, Germany).

### Statistical analysis

For statistical analysis of CDRs and viability, mean values were compared by one-way ANOVA followed by Games-Howell’s post hoc test using SPSS software (IBM SPSS V.26.0, SPSS Inc., Armonk, NY; significance level α = 0.05).

## Results

### Microclimate at the peat bog “Saumoos”

**Fig. 2:**
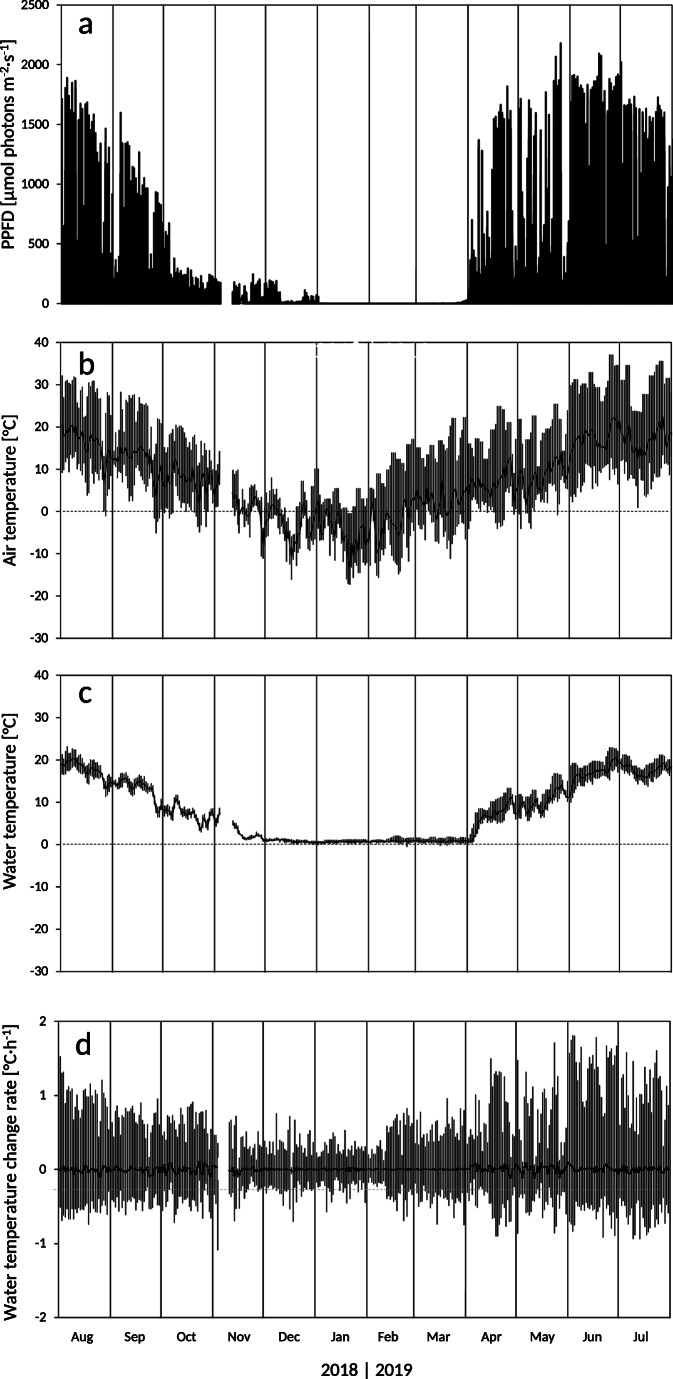
Microclimate (August 1st, 2018, to July 31st, 2019) as recorded at the peat bog “Saumoos” (1065 m a.s.l.) near St. Margarethen, Tamsweg, Salzburg, Austria. **a** Photosynthetic photon flux density (PPFD, 5 cm above the water level), **b**–**d** daily maximum (upper bars), minimum (lower bars) and mean (black line) air temperature 2 m above the ground surface (**b**), water temperature 8 ± 2 cm below surface of a peat bog pool inhabiting *Micrasterias* cells (**c**) and rate of change in water temperature (**d**). Measurement interval: 30 min

Solar irradiation on the water surface showed an annual course typical for this elevation in central Europe with a summer maximum value of 2180 μmol photons m^-2^·s^-1^ (Fig. 2a). After the formation of a compact winter snow cover at the beginning of January 2019, irradiation dropped to zero for the duration of 3 and a half months, i.e. *Micrasterias* experienced complete darkness during the winter.

While the air temperature (Fig. 2b) reached +37.2 °C in summer and −17.3 °C in winter, water temperature in the peat bog pond (Fig. 2c) was more moderate with a temperature maximum and minimum of +23.2 °C in summer and −0.6 °C in winter, respectively. The annual and daily amplitudes of the water temperature were also considerably lower. In the course of the year, the water temperature varied only by 23.8 °C which is less than half of the 54.5 °C air temperature variation. The maximum daily amplitude during the summer months (June to August) did not exceed 6.5 °C and was with 2.0 °C much lower in winter (December to February). In comparison, the air temperature showed maximum daily amplitudes of 27.6 °C in summer and 26.7 °C in winter. On 40.3% of the measurement days, daily mean water temperatures were lower than +4 °C.

The water body predominantly remained unfrozen. During winter, the water surface was covered by a thin layer of ice and on top by a blanket of snow (up to approximately 1 m). From December until March, *Micrasterias* experienced hardly any change in temperature. The water body temperature was mostly close to +0.8 ± 0.2 °C (mean ± SD) and varied only between −0.6 °C and +2.1 °C. In line with this, the rates at which water temperature changed were generally low but slower in winter (−0.7 to +1.0 °C∙h^-1^) than in summer (−1.0 to +1.8 °C∙h^-1^; Fig. 2d).

### Effect of temperature on cell division rates

**Fig. 3:**
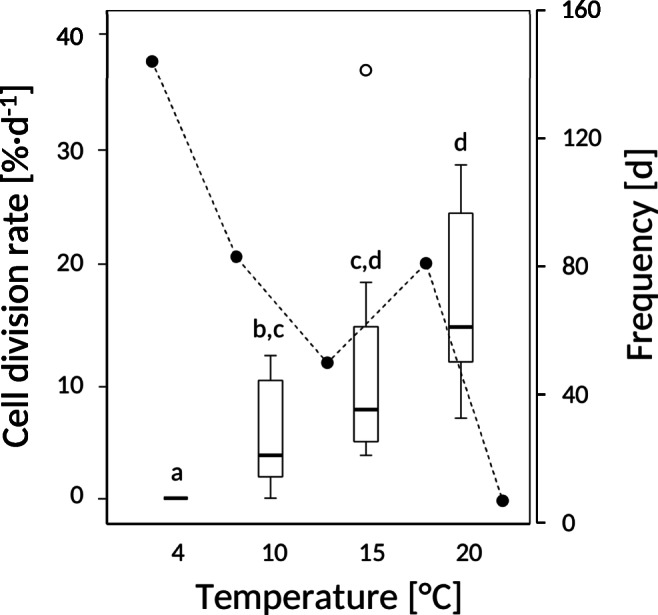
Cell division rates (CDR) as affected by cultivation temperatures. Boxes represent the interquartile range (IQR) containing 50% of the data: horizontal line inside the box, median; whiskers, minimum and maximum; open circle, outlier (value between 1.5 and 3 times IQR). Lower case letters indicate significant differences between mean values (one-way ANOVA followed by Games-Howell’s post hoc test, P < 0.05). Additionally, solid circles indicate the absolute frequencies of the water temperatures measured at the “Saumoos” site (2018–2019)

Low growth temperatures significantly impeded cell division rates (CDR) of *Micrasterias*. CDR increased with increasing temperatures from zero at +4 °C to a mean value of 16.6 %∙d^-1^ at +20 °C (Fig. 3). While at +4 °C cell division was completely inhibited, at +10 °C on average, 1–2 divisions were counted per day; at +15 °C, the number of cell divisions reached half of the control value (+ 20 °C). In nature at the “Saumoos” site on 144 days (40.3%), the water temperatures were < +4 °C. Temperatures ≥ +4 °C and < +10 °C were recorded on 83 days (21 %), temperatures ≥ +10 °C and < +15 °C on 50 days (14 %) and temperatures ≥ +15 °C and < +20 °C on 81 days (22.7 %). Water temperatures > +20 °C were only observed on 7 days (2 %).

### Freezing resistance and tolerance to ice encasement

**Fig. 4:**
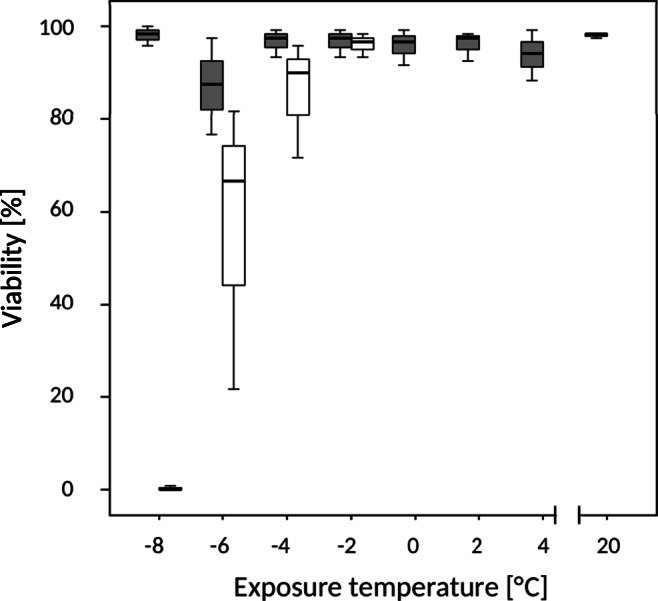
Effect of ice encasement during low-temperature exposure for 1 h on viability of *Micrasterias*. During low-temperature exposure, ice formation was either inhibited (grey boxes) or artificially induced at the respective target temperature (white boxes). The number of surviving cells was then counted, and the viability was expressed as surviving cells in percentage of total. Cooling from +4 °C to the different target temperatures was performed at a moderate rate of −2 °C∙h^-1^. Boxes represent the interquartile range (IQR) containing 50% of the data; the horizontal line inside the box is the median. Whiskers reach to the minimum and the maximum value. Open circles mark outlier values between 1.5 and 3 times of the IQR

Viability of *Micrasterias* cells was fully unaffected by exposure to low non-freezing temperatures. After 21 days of exposure to +4, +10 and +15 °C, cell viability was in the same range as in untreated controls exposed at +20 °C (average 96%; Fig. 4; grey boxes). *Micrasterias* cells also readily survived exposure to sub-zero temperatures if the medium was kept supercooled and free of ice: cell viability remained above 90% even if the cells got subjected to a temperature of −8 °C (cooling rate, −2 °C∙h^-1^) and were rewarmed at +20 °C (Fig. 4; grey boxes). Survival was completely different when freezing temperatures were accompanied by ice encasement. Already after 1 h and exposure to −4 °C, approximately 10% of the cells had died. With decreasing freezing temperature, damage increased successively. Exposure to −6 °C was lethal for ~40% and to −8 °C for ~95% of cells (Fig. 4; white boxes).

**Fig. 5:**
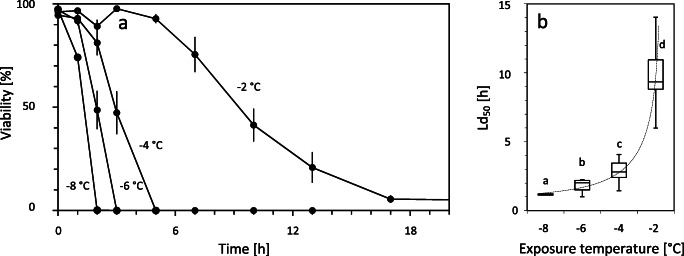
Effect of freezing temperature and duration of ice encasement on the survival of *Micrasterias* cells. Cell suspensions containing culture medium and algal cells were frozen to different temperatures. After thawing, in regular intervals, the number of vital cells was assessed. **a** Time course of the viability, i.e. the percentage of cells still alive. Data points represent mean values (± SE) of 15 parallels each containing 40 cells. **b** Lethal duration (Ld_50_) indicates the time span after which 50% of the individuals died at the respective freezing temperature when encased in ice. Boxes, median, upper and lower quartile; whiskers, minimum, maximum value. Significant differences are indicated by different letters (one-way ANOVA followed by Games-Howell’s post hoc test, P < 0.01)

While ice encasement was partly survived by *Micrasterias* cells when it lasted for a relative short time period, prolongation of exposure became rapidly lethal (Fig. 5a). At −4, −6 and −8 °C, it took between 2 and 5 h until it was lethal for the cells. Under a more moderate freezing temperature of −2 °C, it took significantly longer until all cells had died. Correspondingly, the exposure time that leads to the death of 50% of the cells (Ld_50_) showed significant temperature dependence (Fig. 5b). Nevertheless, after 123 h at −2 °C, 2% of cells were still alive.

### *Micrasterias* does not frost harden

**Fig. 6:**
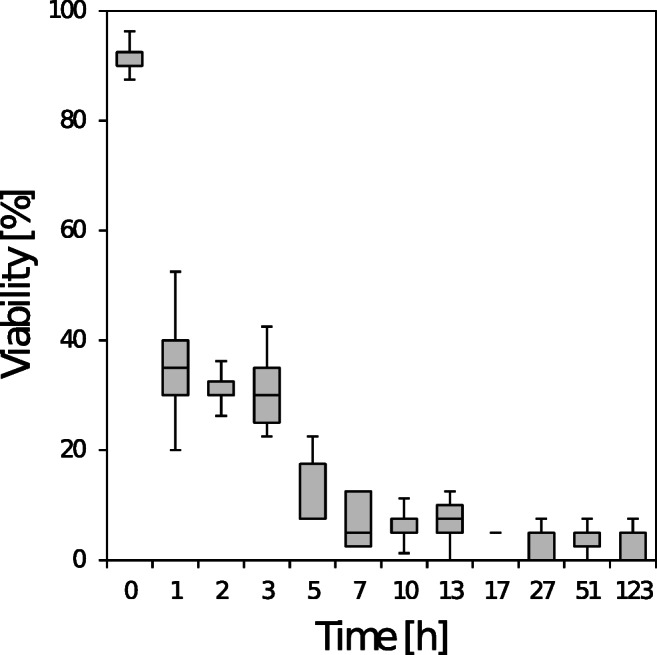
Susceptibility of *Micrasterias* cells to ice encasement after a controlled cold acclimation treatment. Subsequent to a 51-day cold acclimation treatment (see Fig. 1), the algae were cooled from +0.5 °C to −2 °C at a rate of −2 °C·h^-1^. Then ice nucleation was induced, and the algae were further kept at −2 °C. At different time intervals, samples from the ice-encased *Micrasterias* cells were thawed, and the percentage of vital individuals was determined using a positive plasmolysis test

*Micrasterias* cells were exposed to an artificial cold acclimation treatment to assess the cold acclimation capacity of the species. In the treatment, the natural autumn-winter slow temperature decrease in the water body of peat bogs (−0.6 °C·d^-1^) and also the change in irradiation climate from a moderate intensity to darkness was simulated (see Fig. 1). After this cold acclimation treatment at day 51, freezing resistance and ice encasement susceptibility of *Micrasterias* cells were tested again by ice nucleation at -2 °C and exposure for different durations (Fig. 6). No improvement, i.e. frost hardening, could be detected when viabilities were compared to non-cold-acclimated *Micrasterias* cells.

### Ultrastructural alterations in experimentally cold acclimated cells in comparison to cells of *Micrasterias* field-sampled in autumn

**Fig. 7:**
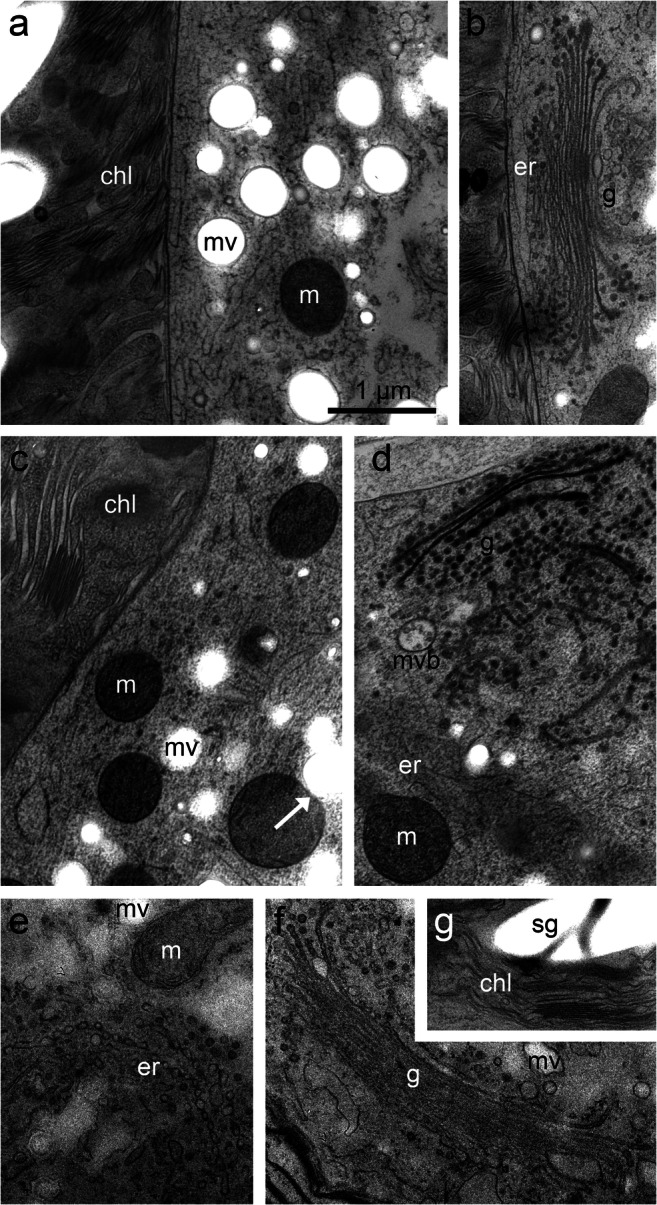
TEM micrographs of *Micrasterias denticulata*. **a**–**b** Untreated control cells (+20 °C) show round, solitary mitochondria (m), mucilage vesicles (mv) and unaltered chloroplasts (chl), Golgi body (g) and ER cisternae (er). **c**–**d** Experimentally cold acclimated cells (+0.5 °C, darkened) show slightly bloated thylakoids of the (chl), round (m), degraded (g), appearance of multivesicular bodies (mvb) and slightly enlarged (er). Interaction of (m) and (mv) is visible (arrow; **c**). **e**–**g** Cells field-sampled in late autumn show slightly elongated (m), an enlarged (er), unaltered (g) and bloated stroma thylakoids and regular starch grain (sg) in the (chl). Scale bar (1 μm) applies to all images

TEM investigations of *Micrasterias* control cells (LC; +20 °C) showed an intact ultrastructure as previously reported (Steiner et al. 2020). The cells contained the round/oval and equally distributed mitochondria, typical for *Micrasterias* (Andosch et al. 2012; Steiner et al. 2018; Steiner et al. 2020). Furthermore, the mucilage vesicles, regular thylakoid membranes, endoplasmic reticulum (ER) and Golgi bodies with a regular number of 11 cisternae and an average vesicle production were visible (Fig. 7a, b). In *Micrasterias* cells exposed to the cold acclimation treatment (51 days; cooling rate, −0.6 °C·d^-1^), to a final temperature of +0.5 °C and exposed to darkness, the interaction of the mitochondria and mucilage vesicles was visible (arrow; Fig. 7c), thylakoid membranes and ER cisternae appeared slightly bloated, and Golgi bodies showed degraded cisternae, fragmented into numerous vesicles with accompanying multivesicular bodies (Fig. 7c, d). Field-sampled *Micrasterias* cells depicted slightly elongated mitochondria and an enlarged ER. Golgi bodies appeared unaltered, and (mucilage) vesicle production was still maintained (Fig. 7e, f). Chloroplasts showed bloated stroma thylakoids, but the “grana” thylakoids were dense (Fig. 7g).

### Distribution of *Micrasterias* during winter

To semi-quantitatively describe the distribution of *Micrasterias* in the peat bog “Saumoos”, the overall diversity of selected desmids during winter was estimated in the field and was categorized from very common to very rare. In deeper sphagnum areas, the following algae were found: *Netrium digitus* (very common), *Micrasterias denticulata* (very rare), *Micrasterias truncata* (very rare), *Micrasterias rotata* (rare), *Euastrum oblongum* (rare) and *Closterium lunula* (very rare). In the insulating ice cap and the surrounding water and peat bog ground, only *Netrium digitus* (very rare) was observed.

## Discussion

The environmental conditions in the microhabitat of *Micrasterias denticulata,* as determined at “Saumoos” in peat bog pools, are such that they allow to escape freezing during the winter period despite severe atmospheric freezing temperatures down to −17.3 °C. In the area of the study site, heavy frosts regularly occur in winter due to frequent temperature inversions. On clear winter nights, air temperatures lower than −20 °C (−28.3 °C from 1971–2000; ZAMG 2020) were often measured. Our data confirm this but also show that *Micrasterias* is in its natural microhabitat is not affected by these low freezing temperatures, being exposed to much more moderate temperatures at the ground of the water body of the peat bog. Large parts of the peat bog pools remained unfrozen and by snow coverage cells got exposed in darkness and at a moderate and stable temperature around +0.8 ± 0.2 (mean ± SD) °C. In principle, this also applies to locations at higher elevations in Tamsweg, Lungau, as during winter 2019/2020, similar mean water temperatures of 0.0 ± 0.1 °C and +0.6 ± 0.2 °C were recorded in the “Weitschober Moor” (1675 m a.s.l.) and the “Überling Moor” (1720 m a.s.l.), respectively. Under these environmental conditions, *Micrasterias* can escape freezing and, thus, as the results of our artificial cold acclimation treatment show, does not — but what is more important has no need to cold acclimate or to become tolerant to ice encasement. In fact, the species does not tolerate ice encasement for an extended period of time which contradicts former believes that the species may survive enclosed inside of ice bodies. “Hibernation” of *Micrasterias denticulata* as a zygospore was also considered, however, after numerous excursions over the years of observations in the peat bog were virtually absent (personal communication U. Lütz-Meindl). *Micrasterias denticulata* surviving winter as a zygospore is therefore rather inconceivable. This might be different for other *Micrasterias* species, e.g. in *M. rotata* zygospores are commonly found (Anissimova and Terlova 2016). However, the strategy to survive winter freezing temperatures in sensu Larcher (1983) is to “escape”, similar to hydrophytes or herbaceous species that outlast in always warmer soil. While the strategy of *Micrasterias* is to escape, other algae have proven to achieve considerable freezing tolerance in winter (Elster et al. 2008; Hawes 1990; Jimel et al. 2020; Trumhova et al. 2019).

Cell division rates (CDR) of *Micrasterias* cells were significantly affected by low temperature and decreased with decreasing temperatures from +20 °C to +4 °C. The zero CDR determined at +4 °C indicates that in nature, cell divisions must be ceased for about 5 months. Recorded water temperatures indicate that cell division may stop in the mid of November and does not restart before the mid of April. Based on our results, high CDRs of 16.6 ± 8%·d^-1^ (mean ± SD) up to 28.9%·d^-1^ can be expected in June, July and August.

*Micrasterias* cells overwinter at moderate positive water temperatures in complete darkness. Darkening is caused by ice and snow layers during winter months. Temperature conditions are more moderate when compared to which temperatures (0 to –5°C) snow-packed evergreen shrubs in the subalpine zone are exposed to (Neuner et al. 1999a). Snow-pack and darkening have a positive effect on photosystem II efficiency; water relations and plants are not forced to frost harden to maximum values (Neuner et al. 1999a; Neuner et al. 1999b). Little is known about the physiological effect of darkening on algae. However, in the desmid *Cosmarium crenatum* exposed to darkness for only 1 week, transcripts associated with photosynthesis, photorespiration and cell wall development were repressed (Mundt et al. 2019). *Cosmarium* sp. served also as a test object for cold stress, and a strain-specific effect on the ultrastructure was observed after prolonged exposure to −0.6 °C for 32 days (at 30 μmol photons m^-2^·s^-1^), with subsequent recovery for 8 days (Stamenkovic et al. 2014). This prolonged freezing treatment resulted in numerous alterations of the ultrastructure, particularly in the chloroplast, but also in other structures like Golgi bodies (Stamenkovic et al. 2014). Freezing stress data in *Cosmarium* sp. and other microalgae have been summarized by Stamenkovic and Hanelt (2017).

In *Micrasterias*, ultrastructural alterations were observed after the cells have been exposed to an artificial cold acclimation treatment or were field-sampled in late autumn. As *Micrasterias* cells were unable to frost harden and unable to become tolerant to ice encasement for a longer period, we interpret these cytological changes to be part of winter stress or a winter resting state. It has to be pointed out that freezing-tolerant Zygnematophyceae like *Zygnema* sp. show the formation of a pre-akinete stage, which tolerates frost events down to a much lower temperature (Trumhova et al. 2019). In *Micrasterias* cells, obvious ultrastructural alterations were the enlargement and increased number of the ER cisternae and the bloating of the thylakoid membranes. *Micrasterias* control cells (LC) never showed these alterations. Structural alterations of the ER have been described as stress hallmarks before (Faso et al. 2009), and it was previously reported that the ER is an important interplay associate in the degradation and reestablishment pathway of Golgi bodies in *Micrasterias* (Lütz-Meindl et al. 2016). Stroma thylakoids were bloated, while “grana” thylakoids appeared dense and unaltered. This could be linked to the photoperiodicity of the alga (see also Pfeifer and Krupinska 2005) and might even trigger prospective ultrastructural adaptations of *Micrasterias* in order to prepare for even lower temperatures. General alterations of the chloroplast have also already been observed and described as indications for cold stress in algae and plant cells before (Steiner et al. 2020; Tanaka et al. 2017).

After the artificial cold acclimation treatment, Golgi bodies appeared to degrade. In comparison, these ultrastructural alterations were only partly observed in the samples of *Micrasterias* taken in autumn from the field. We assume that after the artificial cold acclimation treatment, *Micrasterias* cells were more stressed than field-sampled cells and therefore were more in need of degradation, reestablishment and vesicle production of the late endocytic and the trafficking pathway. The large Golgi bodies of *Micrasterias* consist of exactly 11 cisternae during the whole cell cycle, and degradation processes were already investigated in *Micrasterias* during various abiotic stresses including cadmium stress (Lütz-Meindl et al. 2016). In contrast, during +4 °C chilling stress (up to 21 d), Golgi bodies appeared unaltered but rapidly degraded to single cisternae when freezing was induced at −2 °C (Steiner et al. 2020). In comparison, our results depict minor degradation processes of Golgi bodies of *Micrasterias* after artificial cold acclimation treatment. It appears that Golgi bodies respond to chilling stress inert in more degradation steps when the cooling time is artificially extended. This was not the case in cells sampled in the field, when Golgi bodies resemble those of LC cells. The number of multivesicular bodies (MVBs) was increasing with decreasing temperatures. As a late part of the endocytic pathway, the MVBs might support the cell by recycling damaged constituents for further reestablishment of proteins and compartments that are essential during low-temperature stress. Furthermore, MVBs are often in close spatial contact with trans Golgi and ER cisternae, indicating that both organelles are essential for the development of the MVBs (Wanner et al. 2013). Nevertheless, MVBs were also only infrequently found in the cells of *Micrasterias* sampled in the field.

*Micrasterias* taken in autumn from the field showed infrequent mitochondrial alterations, such as elongation*.* But even there, mitochondrial alterations were not as pronounced as described by Steiner et al. (2020), where *Micrasterias* cells showed mitochondrial fusions in response to short-term low-temperature exposure (+4 °C) and subsequent freezing stress (Steiner et al. 2020).

The variety of vesicles in *Micrasterias* has been extensively studied before (Meindl et al. 1992). Amongst the different vesicle populations, mucilage vesicles appear to be of major importance for movement and protection of *Micrasterias* (Aichinger and Lütz-Meindl 2005). In Steiner et al. (2020), it was visualized that the interactions of mitochondria and peroxisomes with mucilage vesicles support the cells during chilling and freezing stress by excreting degraded cell constituents to the cell surface. This was partly validated in the present study by the interaction of mucilage vesicles with mitochondria, suggesting a similar function. Moreover, *Micrasterias* cells are capable of directional movement via local mucilage excretion (Oertel et al. 2004), which helps the cells to avoid unfavourable environmental conditions such as freezing by escaping into deeper peat bog zones. This was also confirmed by our on-site field sampling during winter months, when no *Micrasterias* cells were observable whether in the water column, nor in the bog area close to the ice or in the insulating icecap. Nevertheless, single *Micrasterias* cells were found, among other Zygnematophyceae, in deeper sphagnum areas of the peat bog during winter.

## Conclusion

In winter, environmental conditions measured in peat bog pools allow *Micrasterias* cells to survive by the “escape” frost survival strategy. Water temperatures above 0 °C throughout winter permit that the species is unable to frost harden and does not tolerate ice encasement. Bloating of the ER and thylakoids in *Micrasterias* cells that were observed both in response to simulated and to natural low temperature in the field are suggested to be involved in metabolic adjustments during cold acclimation. The distinct differences in the Golgi apparatus and the pronounced vesicle production of the field-sampled cells could facilitate direct movement of cells by vesicle excretion in order to overwinter in deeper layers of peat bog pools seeking shelter from contact to ice.

## Data Availability

Primary data can be provided on request.
